# Hydroxyurea mobile directly observed therapy versus standard monitoring in patients with sickle cell anemia: a phase 2 randomized trial

**DOI:** 10.1038/s43856-024-00552-5

**Published:** 2024-08-09

**Authors:** Philip Sasi, Abel Makubi, Raphael Z. Sangeda, Mariam Y. Ngaeje, Bruno P. Mmbando, Joseph Soka, Caterina Rosano, Alex S. Magesa, Sharon E. Cox, Julie Makani, Enrico M. Novelli

**Affiliations:** 1 Sickle Cell Programme, Muhimbili University of Health and Allied Sciences, Dar es Salaam, Tanzania; 2 Department of Clinical Pharmacology, School of Biomedical Sciences, Muhimbili University of Health and Allied Sciences, Dar es Salaam, Tanzania; 3 National Institute for Medical Research, Tanga Cente, Tanga, Tanzania; 4 Graduate School of Public Health, Department of Epidemiology, University of Pittsburgh, Pittsburgh, USA; 5 Muhimbili National Hospital, Central Pathology Laboratory, Dar es Salaam, Tanzania; 6 Department of Curative Services, Ministry of Health, Dodoma, Tanzania; 7 School of Tropical Medicine and Global Health, Nagasaki University, Nagasaki, Japan; 8 Institute of Tropical Medicine, Nagasaki University, Nagasaki, Japan; 9 London School of Hygiene & Tropical Medicine, London, UK; 10 SickleInAfrica Clinical Coordinating Center (CCC), Dar es Salaam, Tanzania; 11 Imperial College London, London, UK; 12 School of Medicine, Department of Medicine, Division of Classical Hematology, University of Pittsburgh, Pittsburgh, USA

**Keywords:** Health care, Haematological diseases

## Abstract

**Background:**

Sickle cell anemia (SCA) prevalence remains high in sub-Saharan Africa. Long-term treatment with hydroxyurea (HU) increases survival, however, poor adherence to treatment could limit effectiveness. Whilst HU treatment adherence is currently high, this might decrease over time.

**Methods:**

We conducted a single-center, randomized, open-label, parallel group phase 2 controlled clinical trial to determine whether mobile Directly Observed Therapy (m-DOT) increases HU treatment adherence (NCT02844673). Eligible participants were adults with homozygous SCA. People on a chronic blood transfusion program, with hemoglobin (Hb) A levels greater than 20% of the total Hb, total Hb less than 4 g/dL, pregnant or HIV positive were excluded. After a 3-month pre-treatment period participants were randomized to either m-DOT or standard monitoring arm. All participants received smart mobile phones and were treated with HU (15 mg/kg) daily for three months. In the m-DOT arm, drug intake was video recorded on cell phone by the participant and the video sent to the study team. The primary objective was to evaluate the effect of m-DOT on adherence to HU treatment by medication possession ratio (MPR).

**Results:**

Of the 86 participants randomized, 76 completed the trial (26.13 ± 6.97 years, 63.5 % female). Adherence was high (MPR > 95 %) in both groups, 29 (80.6 %) in m-DOT versus 37 (94.9 %) in the standard monitoring arm (P = 0.079). No HU treatment was withheld from participants due to safety concerns.

**Conclusions:**

m-DOT did not increase adherence to HU treatment. We recommend that further testing in larger trials with a longer follow up period be undertaken.

## Plain language summary

Sickle cell anemia (SCA) is an inherited blood disorder in which there is an abnormal protein inside red blood cells. This results in red blood cells becoming sickle shaped and more easily destroyed in the body. Long-term treatment with hydroxyurea can reduce the frequency of illness and hospitalization. However, often people do not manage to take their medication regularly when treatment is long-term. We therefore investigated whether people with SCA in sub-Saharan Africa are more likely to take hydroxyurea when they are remotely monitored than when they are not. Remote monitoring did not improve adherence. However, our study is small and was undertaken over a short time period when hydroxyurea had only recently become available to people with SCA. We propose further studies, to see if remote monitoring increases medication adherence in people with SCA in other scenarios.

## Abstract (web summary)

Sasi et al. evaluate whether using a smartphone to record drug treatment improves monitoring of adherence to medication and improved treatment outcome. In a pilot study of sickle cell anemia in patients starting long-term treatment with hydroxyurea, adherence was not increased when mobile Directly Observed Therapy (m-DOT) was used.

## Introduction

The global health burden of hemoglobinopathies, including sickle cell anemia (SCA), is comparable to that of infectious and other major diseases^1,2^. Every year, about 300,000 children are born with SCA in the world, 75% of whom are born in sub-Saharan Africa^3^. In Tanzania, between 8000 and 11,000 children are born with SCA annually, one of the highest annual SCA birth rates in the world^4^. Such high birth rates imply that a significant number of people in Tanzania and the rest of sub-Saharan Africa suffer the negative impact of this chronic, debilitating disease on their physical and mental health throughout their lifespan, with the majority of them not surviving to adulthood^5^.

Although survival for children with SCA in the US has improved during the last 30 years^6^, SCA mortality still contributes up to 16% of under-five mortality in Africa^7^. However, significant reductions in mortality and high survival rates have been reported where early diagnosis and comprehensive treatment have been implemented^8,9^. Comprehensive treatments include prophylaxis for infectious complications, long-term use of disease-modifying medications such as hydroxyurea (HU) or L-glutamine, and chronic blood transfusion programs. Curative therapies for SCA such as hematopoietic stem cell transplantation and gene therapy are still not well established and/or remain unaffordable for patients in low-and mid-income countries.

HU has been demonstrated in placebo-controlled multi-center clinical trials to be efficacious in reducing complications such as vaso-occlusive pain crises and acute chest syndrome in children and adults with SCA and in improving survival in adults^10,11^. While there has been strong evidence for the benefit of HU therapy in adults with SCA for many years^12^, more recent safety and efficacy data support the use of this therapy even in very young children^11^. In addition, patients who are adherent to treatment have reduced healthcare utilization^13–16^. The drug is given in a single daily dose and is very well tolerated^11^. A recent multi-center study in sub-Saharan Africa has shown that long-term HU use may be feasible, safe and effective in patients with SCA. In that study, HU reduced the incidence of vaso-occlusive events, infections, malaria, blood transfusions, and death^17^. These data highlight the need for wider access to treatment with HU for patients with SCA.

Experience from the US, where HU is widely available and has been in use for SCA for decades shows that poor adherence is one of the major barriers to effective HU treatment in SCA. Overall adherence to HU as low as mean medication possession ratio (MPR) of 60% among patients with SCA^18^ and overall adherence to daily medications (mean MPR) of 58.4% among children with SCA^19^ have been reported. HU for SCA is a long-term treatment, it is a costly medication in certain low income settings and is not widely available in many African countries. In recent years, however, through National Health Insurance schemes, HU treatment has become part of the standard of care for SCA in some sub-Saharan African countries. Published trials in Africa so far show high adherence scores^20,21^ suggesting that adherence to HU treatment among SCA patients is not a problem in sub-Saharan Africa at the moment. In real life, however, adherence to drug treatment is dynamic and may change over time. A parallel can be drawn with adherence to other medications for chronic non-communicable diseases such as hypertension^22^ and type 2 diabetes mellitus^23^, which remains consistently low. The causes of lack of adherence may be provider-related, patient-related, or system-related. Patient-related barriers to adherence include time and transportation to a clinic and to a pharmacy to obtain refills^15^. Over 20% of families refuse HU treatment because of reasons such as fear of cancer or other side effects, concern about lack of efficacy and unwillingness to take the medicine or attend clinic or go to pharmacy^24,25^. Family-reported barriers include difficulty in obtaining refills from the pharmacy and coming to the clinic for follow-up^15^, fear of cancer and other side effects, not wanting to have required laboratory monitoring, or not thinking the medication would work. Potential barriers to adherence in sub-Saharan Africa have not yet been investigated, but access to care is anticipated to be a major problem given the challenges with transportation to SCA centers for patients who live in rural areas and the limited number of SCA providers.

Approaches that seek to address the determinants of adherence such as DOT have demonstrated improvement in adherence and patient-centered outcomes in other diseases^26^. DOT has been shown to improve adherence in multiple clinical trials in tuberculosis and HIV-AIDS^27–29^. DOT is more than supervised pill swallowing; it is also a means to provide support and education.

Electronic medication adherence monitoring systems or digital adherence technologies such as medication event monitoring system (MEMS caps) have been evaluated extensively in clinical trials for improving medication adherence^28^. These systems can provide useful information on adherence on a long-term basis, however, they are proprietary, relatively expensive, cumbersome to carry and may need to be duplicated for each medication taken by the patient.

A study conducted by Creary et al.^30^ in 15 individuals aged 1–22 years in an urban setting in the US demonstrated that mobile-DOT (m-DOT), where the patient records a video of herself in the act of ingesting the HU pill(s) and sends it to the study team, is feasible, acceptable and can achieve high HU adherence. Median MPR before intervention (0.75) improved to 0.91 and the overall median HU adherence with electronic DOT was 93.3%. Since adherence barriers can vary by age, further studies in other age groups and different geographical settings might expand our knowledge of the impact of m-DOT on HU adherence.

We conducted a randomized controlled trial to eventuate the impact of m-DOT on adherence to HU treatment in adult HbSS patients in Tanzania to see whether the use of this innovative patient care tool is associated with increased adherence.

## Methods

### Trial design and participants

This was a single-center, stratified (Hb < and ≥ 6 g/dL) with balanced randomization (1:1), open-label, parallel group, phase II drug trial conducted at Muhimbili National Hospital (MNH) in Dar es Salaam, Tanzania. Participants were recruited from among patients with SCA registered at the Muhimbili Sickle Cell Clinic and were invited to participate during their routine clinic visits. The clinic attends from 30 to 60 patients per week, on average. The protocol of our study was reviewed and approved by the Muhimbili University of Health and Allied Sciences (MUHAS) IRB and the National Health Research Ethics Committee (NatHREC). The detailed design and methods of this study have been published elsewhere^2^. In that publication, the trial was described as a phase 4 clinical trial on the account that hydroxyurea is not a new drug. However, the trial was not designed to address the critical role of phase 4 trials: understanding long-term safety and efficacy in diverse patient populations over extended periods, and in later reviews, a consensus that this was a phase 2 trial was reached. The trial had four stages: enrollment (2 months), pre-treatment follow up (3 months), treatment (3 months), and post-treatment follow-up (2 months). Potential participants were screened for eligibility after giving a written consent. Eligible participants were all adults (aged 18 years or above and living in urban Dar es Salaam), male or female (post-menopausal, sterile, or using an acceptable method of contraception, negative urine pregnancy test at screening and prior to randomization and dosing) with HbSS genotype, absolute neutrophil count > 1500/µL, platelet count > 95,000/µL, serum creatinine < 100 µmol/L, alanine aminotransferase (ALT) less than two times the upper limit of normal; and being able to record and submit videos electronically. Patients on a chronic blood transfusion program as defined by participating in a scheduled (pre-planned) series of transfusions for prophylactic purposes or with a hemoglobin A level that is > 20% of the total hemoglobin; hemoglobin < 4.0 g/dL, HIV positive were excluded. Also excluded were female patients who were planning to conceive during the study period; patients with serious mental (including psychosis) or physical illness, which in the opinion of the investigators, would compromise participation in the study (e.g. impaired mental capacity, alcoholism) and patients with any condition that the investigators would judge to preclude safe participation in the study or to confound the evaluation of the study outcome. Eligible participants were randomized to either the m-DOT arm or the Standard Monitoring arm.

### Interventions

Participants in the m-DOT arm received smart mobile phones, HU therapy (15 mg/kg, Cipla 500 mg tablets, Cipla Ltd, Mumbai, India) and their drug intake was monitored through m-DOT. For each drug intake, they received reminders on their mobile phones at pre-arranged times; and during medication intake they self-recorded a continuous uninterrupted video of themselves, which included a statement of their study ID number and the current date, a clear view of the HU tablets before they were swallowed, a view of the participant swallowing HU, and a view of the participant opening their mouth after they had swallowed HU. The video was then uploaded to WhatsApp messenger (WhatsApp Inc., Mountain View, CA, USA) and sent to the study coordinator’s mobile phone for storage in REDCap. Participants on the standard monitoring arm also received smart mobile phones, and HU therapy at the same dose, but they did not receive medication intake reminders and they neither self-recorded videos of their drug intake nor sent videos to the study coordinator. Participants in both groups were followed up at 2 weeks after initiation of therapy and monthly thereafter through study clinic visits and at each visit were assessed for adherence, response to treatment and safety outcomes. They were also contacted daily through phone calls or text messages to check for the presence or absence of sickle cell-related symptoms including fever, clinic visits or hospitalization at other hospitals.

Adverse events (AEs) related to the administration of HU were monitored according to standard clinical practice. All serious AEs (SAEs) and non-serious AEs were followed up until resolution or until the investigator and the trial clinician agreed that the AE/SAE had stabilized and no more follow-up was required.

During the study, a therapeutic partnership between participants in the m-DOT arm and study team members was established and maintained through cell phone text/call reminders, positive feedback and face-to-face contact during follow-up clinic visits. Additionally, a WhatsApp group of all participants and the study coordinator was formed where they could ask questions or post queries that were addressed by the study team, through the study coordinator, and any relevant information was shared. Participants were compensated for travel costs and HU was provided free of charge. In both arms, HU treatment was not continued beyond the three-month treatment phase of the study.

### Outcomes

The primary endpoint was the proportion of participants achieving ≥ 80% HU adherence as measured by medication possession ratio (MPR) at the end of 3 months of treatment and monitoring. MPR is an indirect way of determining whether a patient is adherent to medication. It is the proportion of days in an observation period that an individual is in possession of a medicine supply. Thus, MPR is calculated by dividing the total days’ supply in the observation period by the number of days in the observation period. Secondary endpoints were the mean change in fetal hemoglobin (%), measured by high-performance liquid chromatography (HPLC, BioRad Variant I) between baseline and the end of three months of HU treatment; the incidence of laboratory (hematology and clinical chemistry) AEs and the incidence of fever and other SCA symptoms through mobile phone-based monitoring, during three months of treatment. Tertiary endpoints included the level of leuκopenia in relation to incidence rates of fever as an indicator of possible infection; mean change in estimated glomerular filtration rate (eGFR), lactate dehydrogenase (LDH) level and reticulocyte count as measures of renal function and hemolysis, respectively. Results for the evaluation of incidence of laboratory adverse events, fever and other SCA symptoms, and level of leukopenia in relation to incidence rates of fever as an indicator of possible infection are not included in this article because the rates of fever were too small for any meaningful analysis.

### Sample size

Based on a study done by Candrilli et al.^18^, we estimated 50 patients per group were necessary to provide an 80% power to detect an estimated proportion with HU adherence of 0.35 in the Standard Monitoring group versus 0.65 in the m-DOT arm, assuming a type I error rate of 5% (*P*-value < 0.05 two-sided) and a 15% drop out rate.

### Randomization

A randomization schedule was generated at a remote site (University of Pittsburgh, USA) and sealed randomization codes were prepared and sent to our center. The randomization was stratified by baseline hemoglobin concentration, just before the start of the treatment period (< 6 g/dL versus ≥ 6 g/dL). The randomization schedule was received and kept by the study pharmacist who was not directly involved in participants’ recruitment or medical care. The allocation of participants to intervention groups was done by the study pharmacist. The investigators were not blinded, but the laboratory technologists performing the analysis of blood tests and the biostatistician conducting the data analysis were blinded.

### Statistical methods

All available data were included in the analysis and there were no data exclusions. Data were analyzed using the following software: IBM SPSS Statistics for Windows, version 26 (IBM Corp. Armonk, N. Y., USA); GraphPad Prism version 9.0; R Core Team (2020); R: A language and environment for statistical computing (R Foundation for statistical computing, Vienna, Austria. https://www.R-project.org/). The proportion of adherence (MPR ≥ 95%) in the two arms was compared using Fisher’s exact test. Median increase in percentage HbF and mean increase in mean corpuscular volume (MCV, difference of the difference), measures of response to HU therapy, were compared using the Mann–Whitney U test.

### Reporting summary

Further information on research design is available in the Nature Portfolio Reporting Summary linked to this article.

## Results

### Participant flow and recruitment

**Fig. 1:**
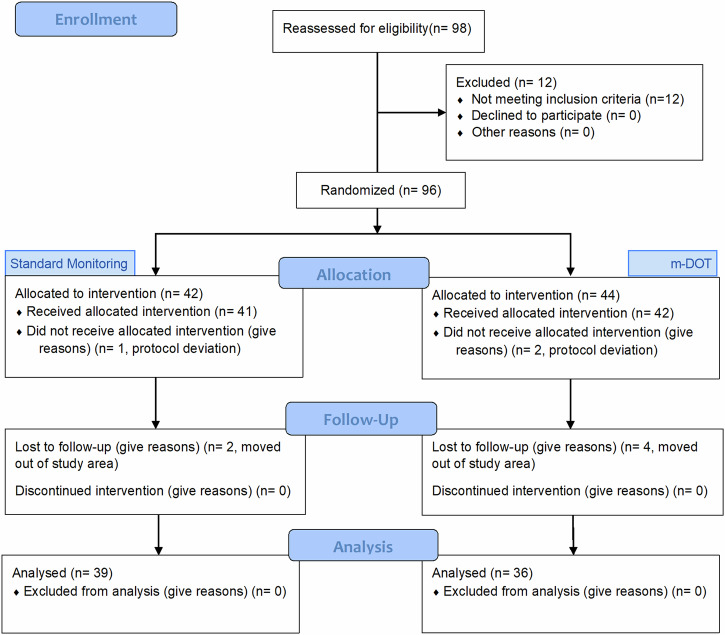
Participant flow and attrition in the trial. CONSORT flow diagram.

A total of 115 patients were screened for eligibility, 105 of whom were eligible. Enrollment, allocation, follow up and analysis of patients who were still eligible at the end of the pre-treatment period are summarized in Fig. 1. The trial was conducted between April 2017 and Feb 2018 and it ended when the last patient in the study completed follow-up. Of the 105 patients enrolled in the study, 98 were still eligible at the end of the pre-treatment period and were reassessed for eligibility of whom 86 were still eligible and were randomized to either of the two arms of the study.

### Baseline data, numbers analyzed, outcomes and estimation, and harms

Of the 86 eligible participants, 75 (87.2%) completed the trial, had adherence data and were included in the final analysis (26.13 ± 6.97 years, 53.3% female, 40 versus 35 who were male). The two arms (m-DOT, *n* = 36; standard, *n* = 39) did not significantly differ in age, sex, education, and MCV (Sysmex XT 2000i, Kobe Japan) or HbF levels at baseline (*P* > 0.1 for all, Table 1).

**Table 1:** Baseline characteristics of study participants

	Treatment arm		
Characteristic	HU m-DOT (Mdn (IQR))	HU standard (Mdn (IQR))	*P*-value
Age (years)	24 (20, 28)	26 (22, 29)	0.243
% female	56.8 (25/44)	54.8 (23/42)	0.788
% single	93.2 (41/44)	88.1 (37/42)	0.297
% with Hb <6 (g/dL)	2.3 (1/44)	9.5 (4/42)	0.101
Reticulocyte count	8.6 (6.9, 12.3)	8.3 (5.9, 11.9)	0.414
LDH (µ/L)	624 (522, 822)	555 (464, 708)	0.062
WBC (Cells/µL) × 10^3^	11.8 (10.1, 14.4)	11.3 (9.0, 12.9)	0.050
Neutrophil (Cells/µL) × 10^3^	6.2 (4.2, 7.3)	5.9 (3.9, 7.1)	0.713
Hb (g/dL)	8.0 (7.2, 9.0)	8.1 (6.9, 8.9)	0.983
HbF (%)	3.8 (1.9, 5.2)	3.2 (1.7, 6.0)	0.368
MCV (fL)	80 (74, 86)	79 (74, 84)	0.563

*HU* hydroxyurea, *m-DOT* mobile directly observed therapy, *Mdn* median, *IQR* interquartile range, *Standard* standard monitoring, *Hb* total hemoglobin, *LDH* lactate dehydrogenase, *WBC* white blood cell count, *HbF* fetal hemoglobin, *MCV* mean corpuscular volume.

**Fig. 2:**
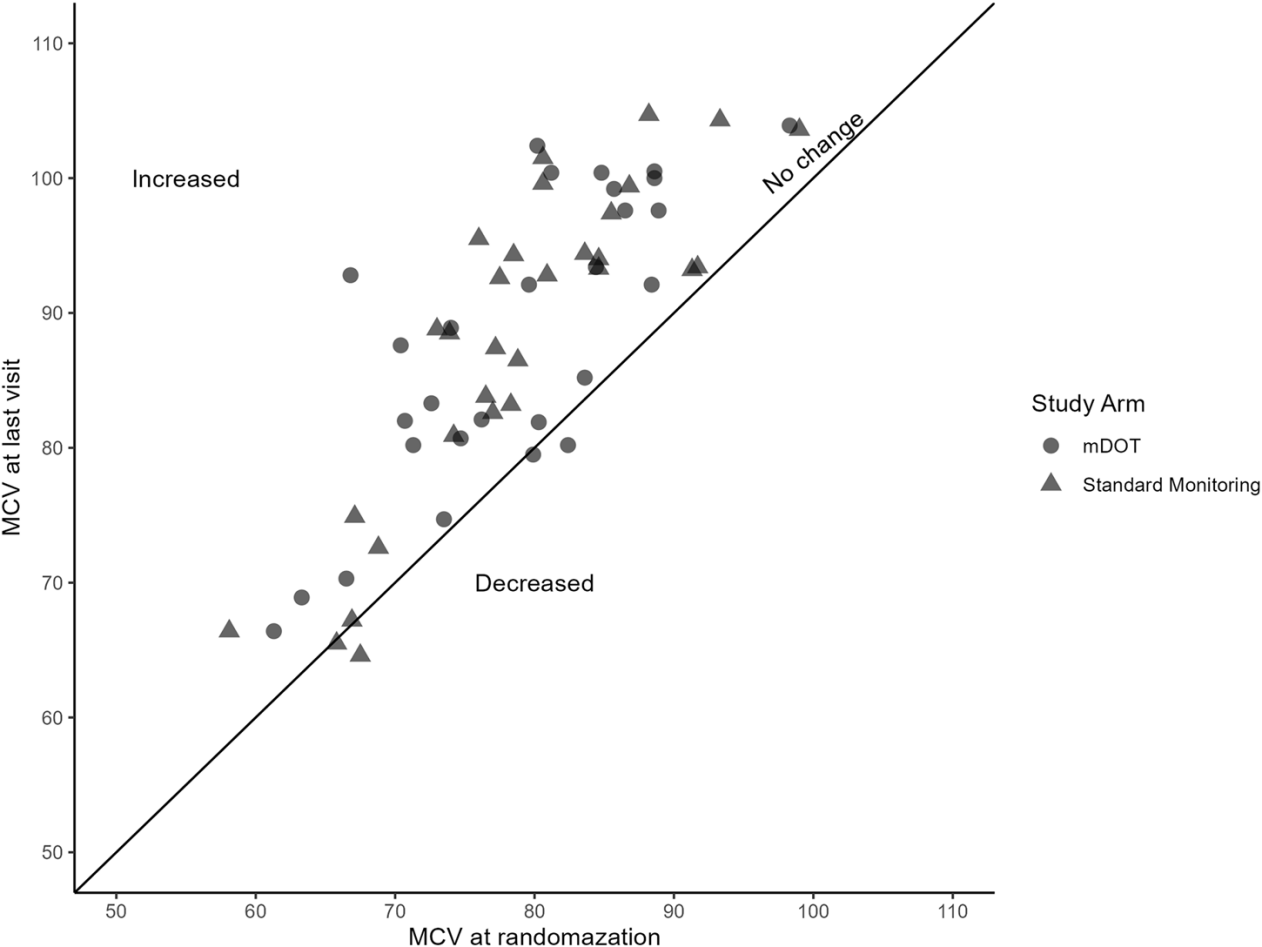
Treatment with HU increases MCV. Scatter plot showing change in MCV after treatment with HU for individual participants (orange, m-DOT arm; blue, standard arm). Source data can be found in Supplementary Data 1

**Fig. 3:**
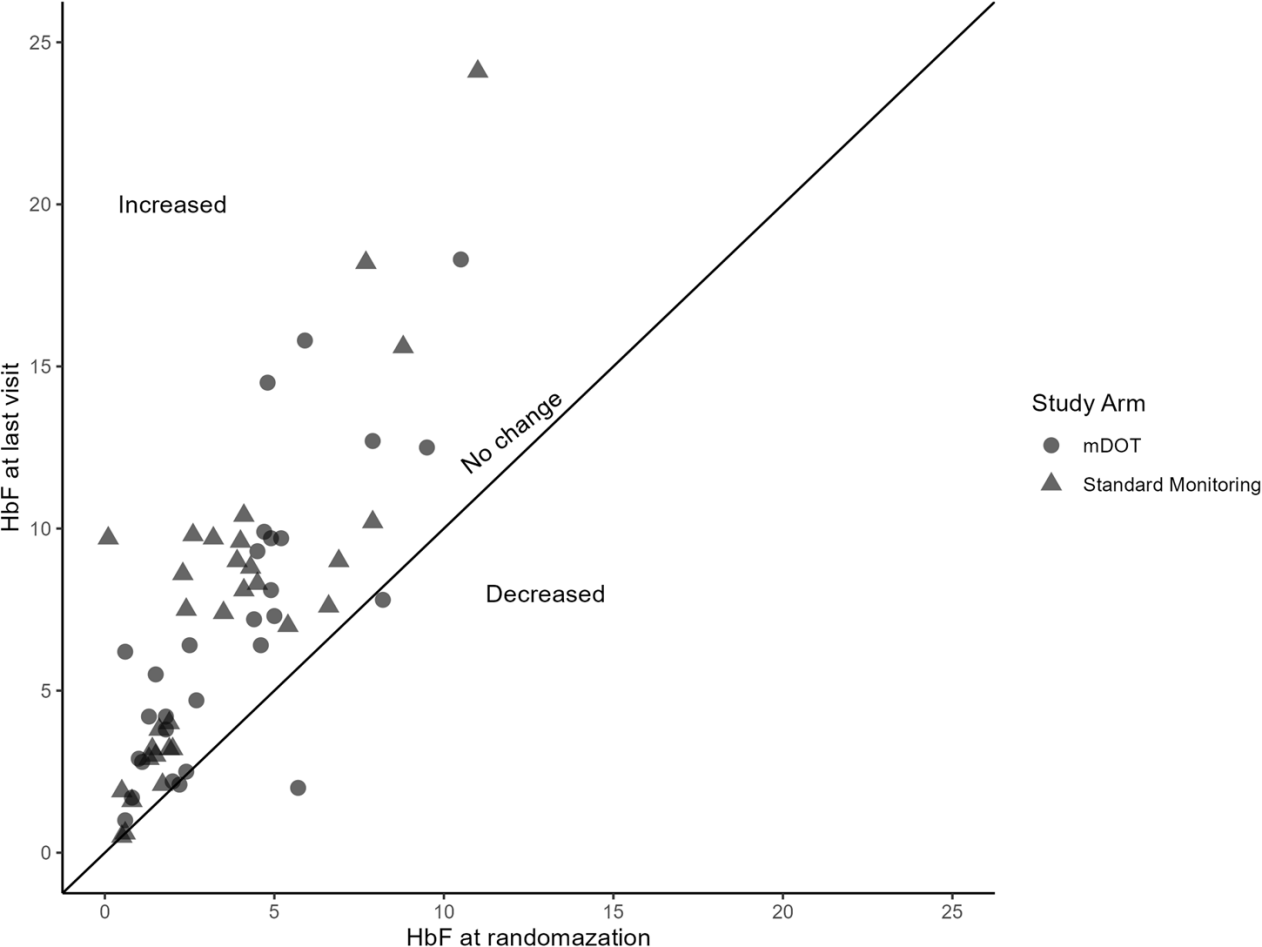
Treatment with HU increases HbF. Scatter plot showing the change in HbF after treatment with HU for individual participants (orange, m-DOT arm; blue, standard arm). Source data can be found in Supplementary Data 2

Adherence was high in both groups and none of the participants had adherence below the cut-off of 80%. There was no difference in the proportion of participants with adherence above 95% between the m-DOT and the standard monitoring arms (29 (80.6%) versus 37 (94.9%); *P* = 0.079). After 3 months of HU treatment, all measured laboratory parameters of long-term clinical importance were not significantly different between the arms (Table 2). Scatter plots showed that the majority of patients in both arms had an increase in MCV and HbF following HU exposure indicating that HU produced the desired therapeutic effect in the majority of patients (Figs. 2, 3, respectively). Disregarding the study arm and analyzing data as if it were a single-arm study, all laboratory parameters changed significantly following HU treatment.

**Table 2:** Adherence and change in laboratory parameters after HU treatment

Parameter	HU m-DOT (Mdn (IQR)) *n* = 36	Standard (Mdn (IQR)) *n* = 39	*P*-value
Adherence (MPR)	1.00 (0.98−1.04)	0.99 (0.98−1.00)	0.147
Reticulocyte count	−3.83 (−7.91, −1.51)	−3.38 (−7.79, −1.20)	0.994
LDH (u/L)	−174.00 (−327.00, −27.00)	−79.00 (−221.25, −8.75)	0.137
WBC (Cells/µL)	−2.92 (−4.57, −1.49)	−3.37 (−4.68, −0.89)	0.863
Neutrophil (Cells/µL)	−1.44 (−3.15, −0.11)	−2.21 (−3.36, −0.68)	0.252
Hb (g/dL)	0.45 (−0.10, 0.90)	0.40 (0.10, 0.80)	0.778
HbF (%)	2.80 (1.30, 4.80)	2.30 (1.40, 6.30)	0.564
MCV (fL)	8.95 (3.78, 13.85)	8.70 (4.60, 14.60)	0.965

*HU* hydroxyurea, *m-DOT* mobile directly observed therapy, *Mdn* median, *IQR* interquartile range, *Standard* standard monitoring, *MPR* medication possession ratio, *Hb* total hemoglobin, *LDH* lactate dehydrogenase, *WBC* white blood cell count, *HbF* fetal hemoglobin, *MCV* mean corpuscular volume.

Three patients had low (< 90 ml/min) estimated glomerular filtration rate (eGFR) at baseline, with two of them improving to normal eGFR at the end of treatment and one patient remaining with low eGFR despite HU treatment. None of the participants had their HU treatment withheld because of safety concerns as outlined by our protocol^2^. Neutropenia (absolute neutrophil count <1500/μL) after treatment was observed in 2 out of 61 (3.3%) of the patients with complete data. There was no significant increase in the number of participants reporting fever or painful episodes after treatment with HU and no patient developed abnormal ALT results after exposure to HU.

## Discussion

### Limitations

Our study has limitations: HU was refilled during monthly study visits and the use of MPR may have overestimated adherence (patients who may have refilled their HU earlier than the scheduled date might have inflated MPR). Like other measures of adherence that summarizes overall adherence with a single number, MPR assumes medication on hand is taken. Additionally, MPR can be insensitive to patient adherence behavior over time.

Participants in our study did not continue HU therapy beyond the three-month treatment phase. While this was an ethical dilemma, it was practically impossible to provide the medication beyond the treatment period. HU treatment for SCA is a life-long treatment, and at the time of the study HU therapy was not part of the standard routine care for SCA in Tanzania, the drug was costly, and not widely available in the country.

### Generalizability

High level of evidence showing clinical and laboratory benefit of HU treatment in patients with SCA has led to HU being incorporated into comprehensive SCA care programs across the world. However, adherence to HU therapy may be the major challenge to HU effectiveness as evidenced by the experience from the US where HU has been in use for decades^18,31,32^. Some African countries have started using HU as part of routine SCA care in recent years and so far studies from this region show that adherence to HU treatment is high. However, adherence to medication is a dynamic process that is influenced by many factors and may change over time.

Like reports from subsequent studies in sub-Saharan Africa, we have observed high adherence on both arms of our study, and higher than previously reported in the US^18^. There are multiple explanations for this observation. First, the drug was only administered for 3 months, which likely mitigated concerns for long-term toxicity, including infertility and leukemogenesis. Second, participants were compensated for the travel costs to and from the sickle cell clinic, and this may have offset transportation as a barrier to obtaining medication refills. Third, some patients may have experienced an improvement in symptoms within a few days of starting HU therapy. This has been described in other studies and may result from placebo effect or from nitric oxide (NO) release from HU molecules^33,34^. Fourth, the drug was given to participants for free and cost was not a barrier to adherence. Finally, increased visibility and empowerment of SCA advocacy in Tanzania through the Sickle Cell Foundation at the time of the study may have been a factor contributing to high adherence. Our findings do not support our prior hypothesis that m-DOT would address patient factors such as lack of adherence to HU intake, fears about side effects, lack of information about the prescribed daily dosage or misconceptions about SCD and treatments, leading to increased adherence. However, we have observed laboratory and clinical benefits from HU administration similar to previous studies despite the relatively short duration of our study. As HU treatment is rolled out across Tanzania, clinical and laboratory benefits, and especially rises in HbF, should be systematically monitored to determine whether maximum benefit is achieved (HbF > 20%)^35^. This will allow timely optimization of therapy, such as switching to dose escalation to the maximum tolerated dose, rather than a clinically effective dose of 15−20 mg/kg per day if maximum benefit is not achieved. In our study, almost all patients did not achieve maximum benefit because our patients were using the lowest recommended dose and the duration of the treatment was short. Maximum benefit is usually achieved at least six months from the start of treatment.

HU administration in our patients was safe. None of the participants had their HU treatment withheld because of safety concerns as prespecified in our protocol^2^. However, reliable evaluation of the effect of HU on renal function was not possible in our study because of the small number of participants with reduced renal function at baseline. In other studies, HU treatment has been shown to improve preexisting renal disease (i.e. GFR, microalbuminuria, urine concentrating ability and renal hypertrophy)^36–38^. It remains unclear whether HU reverses SCA nephropathy and this is an area for further research. A previous study in Uganda^39^ did not find an association between HU treatment and increased incidence or severity of clinical malaria events, the commonest cause of fever in Sub-Saharan Africa, while another study showed a protective effect of HU^17^. Even so, it is important that registries of HU treatment in patients with SCA are established when routine treatment is rolled out in order to appropriately monitor long-term safety in relation to malaria and other infectious pathogens.

### Interpretation

Our study shows that the adoption of mobile technology using cellular phones, now widely available worldwide, can be implemented in sub-Saharan Africa. However, it may not increase the level of adherence to HU treatment during the initial phase of therapy. This may be partly because patients are still enthusiastic about the treatment and are excited by the positive initial response and thus at this stage, the overall adherence is probably at its highest level. However, adherence behavior of patients may change over time. Thus, remote monitoring and m-DOT need further testing, particularly during the later stages of implementing comprehensive routine SCA care that includes HU therapy. Our data also show that a short course of HU therapy for SCA in sub-Saharan is associated with clinical and laboratory benefits and an acceptable safety profile, in accordance with other studies^17^.

In summary, m-DOT may not be associated with increased adherence to HU therapy in SCA in sub-Saharan Africa. However, since adherence is dynamic, the effect of m-DOT needs further testing especially during the later stages of implementation of HU therapy for SCA in the region and by using more robust measures of adherence such as group-based trajectory modeling (GBTM)^40^.

### Supplementary information

Description of Additional Supplementary Files

Supplementary Data 1

Supplementary Data 2

Reporting Summary

## Data Availability

Source data for the main figures in the manuscript are provided in Supplementary Data Files 1 and 2. Other data obtained and/or analyzed during the study are not freely available as they include confidential participant data however are available from the corresponding author on reasonable request.
