# Supplementary material for: Hydroxyurea mobile directly observed therapy versus standard monitoring in patients with sickle cell anemia: a phase 2 randomized trial
Source: Commun Med (Lond). 2024 Aug 9;4:160. doi: 10.1038/s43856-024-00552-5 (PMC11315961; doi:10.1038/s43856-024-00552-5)
Supplement: Supplementary file 1 — Description of Additional Supplementary Files [file 43856_2024_552_MOESM1_ESM.pdf]

Description of Additional Supplementary Files

File name: Supplementary Data File 1

Description: Figure 2: Change in mean corpuscular volume (MCV) after treatment with HU

File name: Supplementary Data File 2

Description: Figure 3: Change in fetal hemoglobin (HbF) percentage after treatment with HU
