# Supplementary material for: Hydroxyurea mobile directly observed therapy versus standard monitoring in patients with sickle cell anemia: a phase 2 randomized trial
Source: Commun Med (Lond). 2024 Aug 9;4:160. doi: 10.1038/s43856-024-00552-5 (PMC11315961; doi:10.1038/s43856-024-00552-5)
Supplement: Supplementary file 4 — Reporting Summary [file 43856_2024_552_MOESM4_ESM.pdf]

nature portfolio

Corresponding author(s): Philip Sasi

Last updated by author(s): Oct 13, 2023

Reporting Summary

Nature Portfolio wishes to improve the reproducibility of the work that we publish. This form provides structure for consistency and transparency in reporting. For further information on Nature Portfolio policies, see our Editorial Policies and the Editorial Policy Checklist.

Statistics

For all statistical analyses, confirm that the following items are present in the figure legend, table legend, main text, or Methods section.

n/a	Confirmed	
☐	☒	The exact sample size (*n*) for each experimental group/condition, given as a discrete number and unit of measurement
☐	☒	A statement on whether measurements were taken from distinct samples or whether the same sample was measured repeatedly
☐	☒	The statistical test(s) used AND whether they are one- or two-sided<br>*Only common tests should be described solely by name; describe more complex techniques in the Methods section.*
☒	☐	A description of all covariates tested
☒	☐	A description of any assumptions or corrections, such as tests of normality and adjustment for multiple comparisons
☐	☒	A full description of the statistical parameters including central tendency (e.g. means) or other basic estimates (e.g. regression coefficient) AND variation (e.g. standard deviation) or associated estimates of uncertainty (e.g. confidence intervals)
☐	☒	For null hypothesis testing, the test statistic (e.g. *F*, *t*, *r*) with confidence intervals, effect sizes, degrees of freedom and *P* value noted<br>*Give P values as exact values whenever suitable.*
☒	☐	For Bayesian analysis, information on the choice of priors and Markov chain Monte Carlo settings
☒	☐	For hierarchical and complex designs, identification of the appropriate level for tests and full reporting of outcomes
☒	☐	Estimates of effect sizes (e.g. Cohen's *d*, Pearson's *r*), indicating how they were calculated

Our web collection on statistics for biologists contains articles on many of the points above.

Software and code

Policy information about availability of computer code

Data collection	No software was used
Data analysis	BM SPSS Statistics for Windows, version 26 (IBM Corp. Armonk, N. Y., USA); GraphPad Prism version 9.0; R Core Team (2020). R A language and environment for statistical computing. R Foundation for statistical computing, Vienna, Austria. https://www.R-project.org/

For manuscripts utilizing custom algorithms or software that are central to the research but not yet described in published literature, software must be made available to editors and reviewers. We strongly encourage code deposition in a community repository (e.g. GitHub). See the Nature Portfolio guidelines for submitting code & software for further information.

Data

Policy information about availability of data

All manuscripts must include a data availability statement. This statement should provide the following information, where applicable:

- Accession codes, unique identifiers, or web links for publicly available datasets
- A description of any restrictions on data availability
- For clinical datasets or third party data, please ensure that the statement adheres to our policy

The datasets generated during and /or analyzed during the current study are available from the corresponding author on reasonable request.

Human research participants

Policy information about studies involving human research participants and Sex and Gender in Research.

Reporting on sex and gender	only sex was considered in the design of our study and during data collection, sex was determined by self-reporting. Data on sex, disaggregated only into female and male were collected. No consent was obtained for sharing individual-level data. of the 75 participants included in the final analysis 48 were female and 28 were male. No sex-based analysis were performed because there was no scientifically plausible reason for possible sex-based differences in the variables that we analyzed.
Population characteristics	Our study involved adult patients (aged 18 years or above) with hemoglobin SS genotype, male or female (post-menopausal, using an acceptable method of contraception, negative urine pregnancy test at screening and negative urine pregnancy test before randomization at start of the treatment period of the study). Participants included in the study also had to have a normal absolute neutrophil and platelet counts; normal renal and liver function.
Recruitment	Participants were recruited from among patients with sickle cell anemia registered at the Muhimbili Sickle Cell Clinic and they were invited to participate during their routine clinic visit.
Ethics oversight	The protocol of our study was reviewed and approved by the Muhimbili University of Health and Allied Sciences IRB and the National Health Research Ethics Committee (NatHREC).

Note that full information on the approval of the study protocol must also be provided in the manuscript.

Field-specific reporting

Please select the one below that is the best fit for your research. If you are not sure, read the appropriate sections before making your selection.

☒ Life sciences ☐ Behavioural & social sciences ☐ Ecological, evolutionary & environmental sciences

For a reference copy of the document with all sections, see nature.com/documents/nr-reporting-summary-flat.pdf

Life sciences study design

All studies must disclose on these points even when the disclosure is negative.

Sample size	A sample size to provide and 80 % power to detect an estimated proportion with HU adherence of 0.35 in the standard monitoring arm versus 0.65 in the mDOT arm, assuming a two-sided type I error rate of 5 % and a 15 % drop out rate, was calculated to be 50 in each arm. The 0.35 proportion of HU adherence was based on a study done by Candrilli et at. in 2011 (Am J Hematol. 86(3): 273-7).
Data exclusions	All available data were included in the analysis and there were no data exclusions.
Replication	We believe that using the design and methods we have described in the manuscript, our data can be reproduced. Testing for replication did not apply in our study.
Randomization	Participants were allocated into the mDOT and the standard monitoring arms of the study using a stratified randomization schedule. This was generated from a remote site using the Research Electronic Data Capture (REDCap) system. Participants were stratified based on baseline hemoglobin concentration (<6g/dL versus > or = 6g/dL) just before the start of the treatment period of the study.
Blinding	Blinding was not relevant to our study because the test intervention was not treatment but the self-recording of a video of drug intake at home. Participants in both arms received same treatment (HU therapy 15mg/Kg/day). It was not possible to blind the sending or not sending of video to the study coordinator. However, the laboratory technologists performing the blood tests and the statistician conducting data analysis were blinded.

Reporting for specific materials, systems and methods

We require information from authors about some types of materials, experimental systems and methods used in many studies. Here, indicate whether each material, system or method listed is relevant to your study. If you are not sure if a list item applies to your research, read the appropriate section before selecting a response.

Materials & experimental systems

n/a	Involved in the study	
☒	☐	Antibodies
☒	☐	Eukaryotic cell lines
☒	☐	Palaeontology and archaeology
☒	☐	Animals and other organisms
☐	☒	Clinical data
☐	☒	Dual use research of concern

Methods

n/a	Involved in the study	
☒	☐	ChIP-seq
☒	☐	Flow cytometry
☒	☐	MRI-based neuroimaging

Clinical data

Policy information about clinical studies

All manuscripts should comply with the ICMJE guidelines for publication of clinical research and a completed CONSORT checklist must be included with all submissions.

Clinical trial registration: Our trial was registered with Clinical Trials.gov (Indentifier: NCT02844673)

Study protocol: Our study protocol is available from the corresponding author on reasonable request and was included with the initial submission

Data collection: Our trial was conducted at Muhimbili National Hospital's Sickle Cell Clinic in Dar es Salaam, Tanzania. Muhimbili National Hospital serves as the main referral hospital in Tanzania. Its Sickle Cell Clinic serves are rendered to 30 to 60 patients per week,on average. Enrollment of patients into the study began in April 2017 and the last patient follow up visit was in February 2018.

Outcomes: The predefined primary outcome was the proportion of participants achieving hydroxyurea treatment adherence (medication possession ratio, MPR > or = 80 % measured at the end of 90 days of medication supply). Secondary outcomes were safety and efficacy of hydroxyurea. Safety was measured by the incidence of clinically significant laboratory adverse events (hematology and clinical chemistry) and incidence of fever and other symptoms reported through daily phone calls. Efficacy was measured by the mean of the change in fetal hemoglobin (%).

Dual use research of concern

Policy information about dual use research of concern

Hazards

Could the accidental, deliberate or reckless misuse of agents or technologies generated in the work, or the application of information presented in the manuscript, pose a threat to:

No	Yes	
☐	☒	Public health
☒	☐	National security
☒	☐	Crops and/or livestock
☒	☐	Ecosystems
☒	☐	Any other significant area

Hazards: Reckless use of smartphones for patient care monitoring or other common uses poses a risk to public health. However, the body of available evidence suggest that the risk is low.

For examples of agents subject to oversight, see the United States Government Policy for Institutional Oversight of Life Sciences Dual Use Research of Concern.

Experiments of concern

Does the work involve any of these experiments of concern:

No	Yes	
☒	☐	Demonstrate how to render a vaccine ineffective
☒	☐	Confer resistance to therapeutically useful antibiotics or antiviral agents
☒	☐	Enhance the virulence of a pathogen or render a nonpathogen virulent
☒	☐	Increase transmissibility of a pathogen
☒	☐	Alter the host range of a pathogen
☒	☐	Enable evasion of diagnostic/detection modalities
☒	☐	Enable the weaponization of a biological agent or toxin
☒	☐	Any other potentially harmful combination of experiments and agents

Precautions and benefits

Biosecurity precautions	No precautions were taken to minimize biosecurity risk during the design or conduct of this trial because there are probably no biosecurity risks associated with our trial. However, the application of the information generated by our trial will need to be accompanied with emphasis on the proper and safe use of the smartphone.
Biosecurity oversight	There are no evaluations or oversights of biosecurity risks of our work that we have received from people or organizations outside our immediate team
Benefits	The use of the smartphone as a patient-monitoring device may improve monitoring of adherence to drug therapy leading to improved treatment outcome and reduced financial burden of implementing conventional directly observed therapy (DOT).
Communication benefits	Benefits of communicating the information generated from our study outweigh the risks because the suggested use of the smartphone is within the normal or common usage. The risk is low and that is why smartphones are now widely used.
